# The Natural History of X-Linked Lymphoproliferative Disease (XLP1): Lessons from a Long-Term Survivor

**DOI:** 10.1155/2020/8841571

**Published:** 2020-08-26

**Authors:** Yike Jiang, Mihail Firan, Sarada L. Nandiwada, Anaid Reyes, Rebecca A. Marsh, Tiphanie P. Vogel, Joud Hajjar

**Affiliations:** ^1^Texas Children's Hospital and Baylor College of Medicine, Department of Pediatrics, Section of Rheumatology, Houston, TX, USA; ^2^Texas Children's Hospital and Baylor College of Medicine, Department of Pathology and Immunology, Houston, TX, USA; ^3^Texas Children's Hospital and Baylor College of Medicine, Department of Pediatrics, Section of Immunology, Allergy and Retrovirology, Houston, TX, USA; ^4^Cincinnati Children's Hospital Medical Center and University of Cincinnati, Department of Pediatrics, Section of Bone Marrow Transplantation and Immune Deficiency, Cincinnati, OH, USA

## Abstract

X-linked lymphoproliferative disease (XLP1) is a rare primary immunodeficiency characterized by EBV-triggered immune dysregulation, lymphoproliferation, dysgammaglobulinemia, and lymphoma. Early childhood mortality from overwhelming inflammation is expected in most patients. The only curative therapy is hematopoietic stem cell transplant (HSCT); however, whether to perform HSCT on an asymptomatic patient remains debatable. This uncertainty arises because the natural history of XLP1 patients without transplantation is not clear. In this case report, we present the natural history of XLP1 in a 43-year-old male patient who did not receive HSCT. We also review the literature on untransplanted XLP1 patients who lived into mid-adulthood. Despite surviving childhood presentations that are typically fatal, we found that these rare patients remain susceptible to manifestations of XLP1 decades later.

## 1. Introduction

X-linked lymphoproliferative disease (XLP1) is a primary immunodeficiency characterized by severe immune dysregulation triggered by viral infection (commonly EBV), hypogammaglobulinemia, and lymphoma. This disease is rare with an estimated incidence of 1–3 in 1 × 10^6^ males [1], a likely underestimate due to the significant risk of early childhood death.

XLP1 is caused by a deficiency in signaling lymphocyte activation molecule- (SLAM-) associated protein (SAP). SLAM family members are signaling receptors broadly expressed on hematopoietic cells. SAP binds to the intracellular domain of SLAM proteins, serving as a molecular switch that toggles between cellular activation and inhibition [2]. Normally, SAP promotes tyrosine phosphorylation of the SLAM receptor and recruitment of downstream signaling molecules, triggering immune cell activation. When SAP is absent in XLP1, these tyrosines on SLAM bind to strong inhibitory molecules that abrogate downstream signaling and activation [3]. The molecular imbalance in SAP deficiency impairs T and NK cell function and abolishes NKT development. Mechanistically, diminished T cell help in germinal centers, compromised immunosurveillance, reduced cytotoxicity, and impaired T cell apoptosis leads to dysgammaglobulinemia, lymphomas, fulminant infectious mononucleosis, and hemophagocytic lymphohistiocytosis (HLH)—the classic features of XLP1 [3].

Diagnosis of XLP1 relies on genetic testing for mutations in *SH2D1A*, but rapid immunologic testing is often critical at the time of presentation. This includes the absence of SAP protein expression by flow cytometry, reduced *ex vivo* NK cell killing, and the absence of NKT cells [4–7].

Since the disease's discovery in 1975 [8], the only curative therapy for XLP1 is hematopoietic stem cell transplant (HSCT). First successfully performed for XLP1 in 1993 [9], HSCT has since significantly improved overall survival [10]. However, there are rare XLP1 patients who remain untransplanted. A few of these patients have survived viral-mediated immune dysregulation early in life and live well into adulthood. The natural history of these patients and reasons for their relative longevity are not well understood. In this case report, we present an XLP1 patient who survived into his forties without HSCT and review the literature for the natural history of XLP1.

## 2. Case Presentation

**Figure 1:**
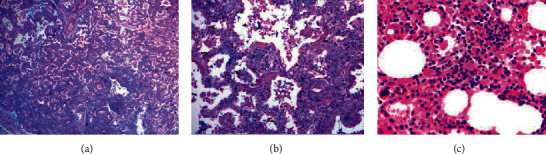
Histopathology during childhood. (a) Lung biopsy at age 6 revealed mature lymphoplasmacytic infiltrate in the alveolar septa consistent with lymphoid interstitial pneumonia. (b) Lung, higher magnification. (c) Bone marrow biopsy at age 12 with erythroid and eosinophilic hyperplasia and granulocytic hypoplasia; hemophagocytosis was absent.

Our patient was healthy and developed normally until age 6, when he exhibited worsening respiratory symptoms. Lung biopsy revealed mature lymphoplasmacytic infiltrate in the alveolar septa consistent with lymphoid interstitial pneumonia (Figures 1(a) and 1(b)). He received corticosteroids and cyclophosphamide for 2 years with significant improvement. At age 12, he developed fever, hepatosplenomegaly, lymphadenopathy, and lymphocytosis consistent with severe infectious mononucleosis. He had a protracted hospital course but eventually recovered without developing HLH (Figure 1(c)) and seroconverted to a typical convalescent pattern. Our patient subsequently developed hypogammaglobulinemia and was started on intravenous immunoglobulin (IVIG).

During the same year, his two brothers (7 and 10 years old) developed fevers and respiratory symptoms and were diagnosed with pulmonary lymphomatoid granulomatosis and hypogammaglobinemia. Despite steroid and cyclophosphamide treatment, one brother succumbed to HLH in the setting of candidemia, while the other died of massive gastrointestinal bleeding. Though XLP1 was considered in the differential diagnosis, it was excluded due to atypical pulmonary manifestations and lack of EBV infections (i.e., negative serologies) in his two brothers [11].

After the death of his siblings, at age 13, our patient experienced recurrent strokes and was found to have necrotizing CNS vasculitis on brain biopsy. He was treated with corticosteroids and interferon-*γ* and recovered with residual left-sided weakness; however, he was lost to follow-up.

**Figure 2:**
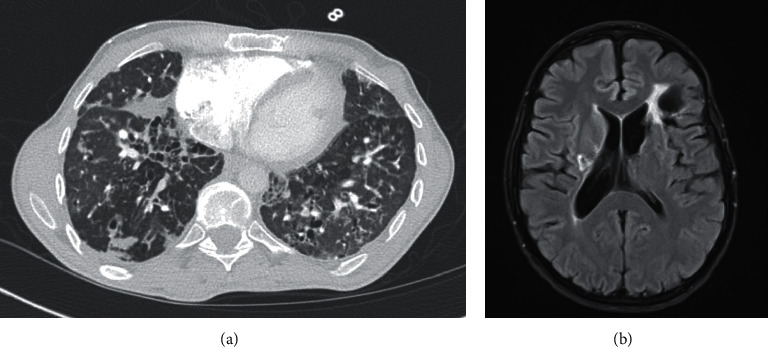
Radiology at age 43. (a) Chest CT scan with diffuse consolidations, bronchiectasis, and cysts. (b) MRI brain showing bilateral areas of acute infarction and left frontal encephalomalacia from prior insult.

In the subsequent years, he retrospectively reported two episodes of shingles, one episode of seizure (age 28), transaminitis (age 37), and a deep venous thrombosis (age 42). He also developed progressive respiratory disease (Figure 2(a)) associated with recurrent pneumonia (1-2 episodes per year). This necessitated oxygen therapy and occasional wheelchair dependence for exercise intolerance, thus limiting his quality of life. He continued to receive monthly IVIG and was started on hydrocortisone for a diagnosis of adrenal insufficiency at age 42.

At age 43, he developed sudden vision change, headache, right-sided weakness, and seizure. MRI of the brain revealed bilateral areas of acute infarction (Figure 2(b)).Additional evaluation failed to identify a primary thrombus. Infectious evaluation was negative for acute infections such as EBV, VZV, CMV, HHV6, and parechovirus, as well as bacteria and parasites. He was initiated on anticoagulation and had complete resolution of weakness to his baseline.

**Figure 3:**
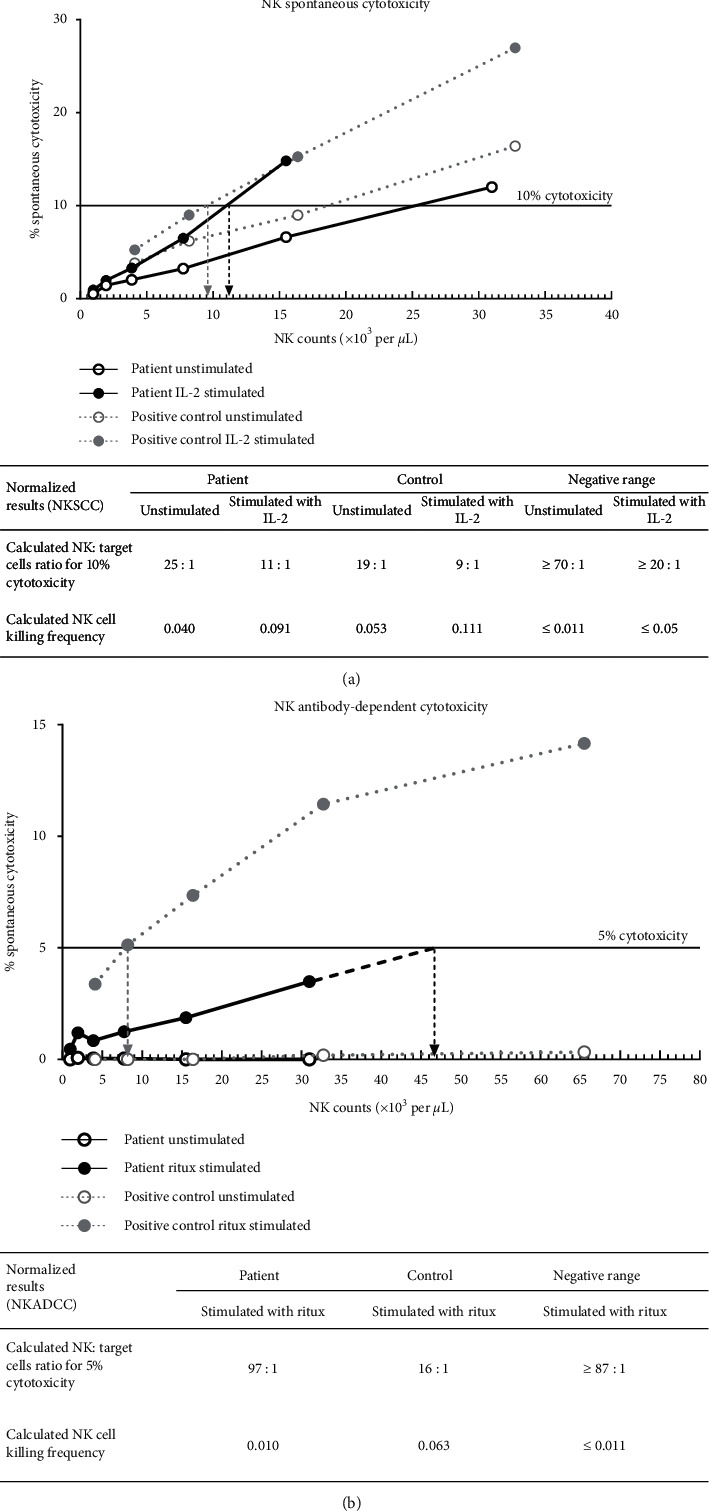
NK cell cytotoxicity at age 43. Peripheral blood mononuclear cells (PBMCs) from healthy controls or the patient were used as effector cells and were incubated with target cells. (a) Target cells were the K562 monocytic cell line lacking MHC class I. Spontaneous cytotoxicity against these target cells was measured following a 4-hour stimulation ± IL-2. In the absence of IL-2, to lyse 10% of target cells, the patient sample required 25 NK cells per one target cell (25 : 1, NK: target cell ratio). Comparatively, the control required 19 NK cells per one target cell (19 : 1). In the presence of IL-2, the patient sample required 11 NK cells per one target cell (11 : 1) and the control required 9 NK cells per one target cell (9 : 1). (b) Target cells were human lymphoblast-like Raji B cells that express MHC class I CD19 and CD20. Antibody-dependent NK cell cytotoxicity against these target cells was measured following a 4-hour stimulation ± rituximab. No cytotoxicity was observed in the absence of rituximab. In the presence of rituximab, to lyse 5% of target cells, the patient sample required 97 NK cells per one target cell (97 : 1), whereas the control required 16 NK cells per one target cell (16 : 1). This test was performed in a CLIA-certified laboratory.

**Figure 4:**
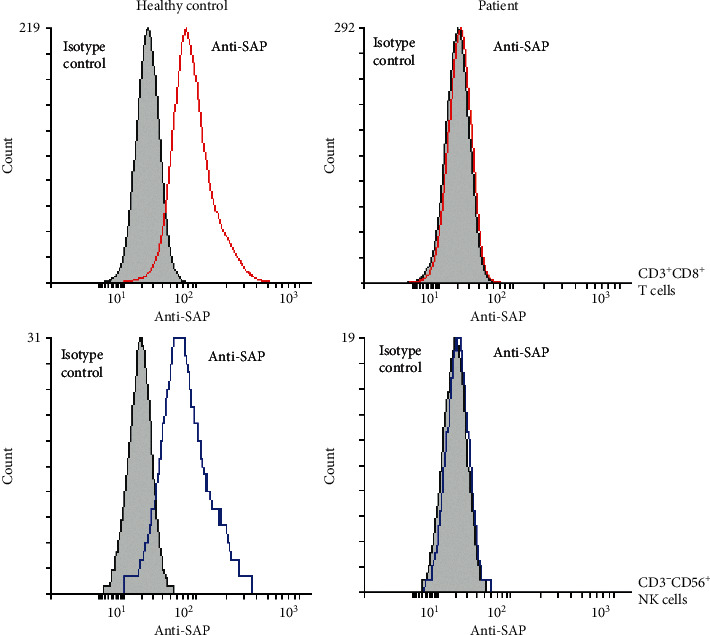
Flow cytometry at age 43. Isotype staining is shaded in gray. SAP staining is shown in color: red in CD3^+^CD8^+^ T cells and blue in CD3^−^CD56^+^ NK cells. A healthy control is shown for reference. This figure is representative of repeated tests on two separate instances as performed in a CLIA-certified laboratory.

**Figure 5:**
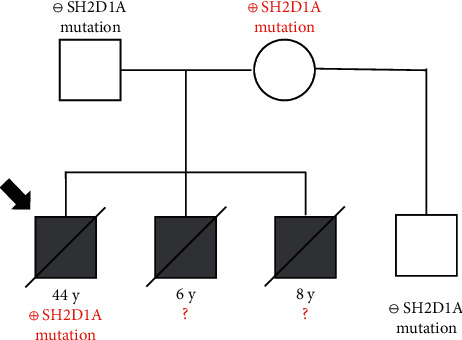
Family history with *SH2D1A* genotype. A full deletion of *SH2D1A* was detected using a commercial immunodeficiency panel (https://www.invitae.com/en/physician/tests/08100/) in the patient and his mother, while his half brother did not have the deletion. The arrow demarcates the case report patient.

Subsequently, the patient was referred to our clinic for reevaluation after 30 years. Immune profiles at this time showed therapeutic IgG troughs and low levels of IgM and IgA. He had normal T cell counts and low B and NK cell counts (Table 1). NK cell cytotoxicity assays revealed normal spontaneous cytotoxicity but decreased antibody-dependent cytotoxicity (Figure 3). By flow cytometry, we found no SAP expression in CD3^+^CD8^+^ T cells and CD3^−^CD56^+^ NK cells (Figure 4). Full deletion of *SH2D1A* was revealed by a commercial immunodeficiency panel (https://www.invitae.com/en/physician/tests/08100/). The patient's mother was a carrier of the same deletion, while his half brother did not have the deletion (Figure 5). Unfortunately, our patient's functional status excluded the option of HSCT.

**Figure 6:**
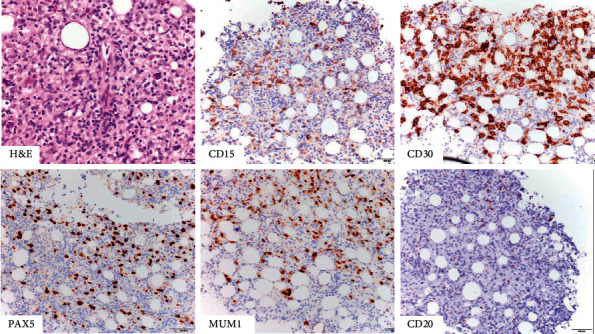
Bone marrow biopsy at age 43. There are large atypical cells on H&E, with a typical staining pattern for classical Hodgkin lymphoma. The neoplastic cells were positive for CD15, CD30, PAX5, and MUM1, while CD20 was negative.

A year after being evaluated at our clinic, our patient developed recurrent pulmonary infections followed by liver failure and pancytopenia. Bone marrow biopsy was consistent with classical Hodgkin Lymphoma (Figure 6). He declined chemotherapy and died a few days after diagnosis.

## 3. Discussion

Although the original presentation of our patient and his brothers at our institution 3 decades ago eluded diagnosis, we were able to confirm our patient's molecular diagnosis of XLP1 using flow cytometry and genetic testing. Retrospectively, our patient displayed most of the classic features of XLP1 in his lifespan including (pulmonary) lymphoproliferation, severe EBV infection, dysgammaglobulinemia, and lymphoma. He did not develop HLH at any stage even when diagnosed with lymphoma at the end of life, and we suspect that this allowed for his survival into adulthood [11].

Although samples of sufficient quality from his siblings were not available for genotyping, we can infer based on their mother's genetic test that they also had whole sequence deletions of *SH2D1A*. Given this assumption, it is noteworthy that the siblings succumbed to XLP1 during childhood, while their brother survived for decades afterwards. This further supports prior observations that XLP1 generally lacks genotype-phenotype correlation [10, 12, 13]. However, it is interesting that all siblings presented with progressive respiratory symptoms due to pulmonary lymphoproliferation that was not associated with EBV infection [11]. Our patient had lymphoid interstitial pneumonia (LIP), characterized by diffuse lymphocytic infiltration of the alveolar septum and pulmonary interstitium. Comparatively, his brothers had lymphomatoid granulomatosis (LYG) which in addition included angiitis and focal necrosis [14, 15]. Though LIP and LYG are viewed as distinct pathological entities, their manifestations in these brothers with presumed syngeneic XLP1 beg speculation as to whether these entities exist on a continuum, especially in the setting of primary immunodeficiency.

Other rare manifestations of XLP1 include gastritis, skin lesions, aplastic anemia, and vasculitis [16–19]. Our patient likely experienced two episodes of CNS vasculitis decades apart. The first was biopsy-confirmed during childhood, and the second was presumed based on clinical presentation and imaging at the age of 43 years. After his latest stroke episode, his neurologic symptoms completely resolved with anticoagulation. We reason that his maintenance IVIG and hydrocortisone provided immunomodulatory effects that aided in his surprising recovery. As exemplified by this case, the majority of reported CNS vasculitis (8/10) in XLP1 has no identifiable infectious trigger [20]. This suggests that CNS vasculitis associated with XLP1 has an intrinsic trigger leading to lymphocytic infiltration into cerebral vessels.

Our case also represents a rare patient with untransplanted XLP1 who lived into mid-adulthood. Literature review revealed six additional untransplanted XLP1 patients who survived into their forties (Table 2) [20–22]. Of the five cases that looked at protein, all had mitigating factors (i.e., genetic reversion or unstable yet functional protein) that led to residual protein expression. Remarkably, our patient survived to a similar age without any SAP expression, further emphasizing the lack of genotype-phenotype correlation. Instead, the shared predictor of relative longevity for these XLP1 patients is that they never developed HLH. The one patient in this group who did develop HLH at age 49 then succumbed to it [22]. Otherwise, the mean age of survival for untransplanted XLP1 patients is 7.5 years with 81.3% mortality from HLH [10].

Even with modern medical advances, XLP1 mortality and morbidity remain high as seen in our patient. Despite surviving life-threatening childhood manifestations and being maintained on lifelong IVIG therapy, he suffered from neurologic deficits and progressive lung disease and eventually died of lymphoma. Similarly, recent published cases of XLP1 patients either do not survive initial childhood manifestations of XLP1 or succumb to complications in mid-adulthood [10, 12, 18, 20–27]. This remains an important point to emphasize during shared decision making with newly diagnosed patients with XLP1 who are eligible for HSCT.

## Figures and Tables

**Table 1:** Clinical immunology profile at age 43.

Immunoglobulin	Levels	(Normal range)	Immune subsets	Total number (103/uL)	(Normal range)
IgE	<2	(≤114 kU/L)	Absolute lymphocytes	1330	4500–11000
IgG	1244	(694–1618 mg/dL)	CD3 + T cells	1250	798–2594
IgM	6	(48–271 mg/dL)	CD19+ B cells	40	63–461
IgA	<5	(81–463 mg/dL)	CD3-CD16+ CD56+ NK cells	39	89–472

The patient had a therapeutic IgG level but low IgM and IgA levels. Flow cytometry also showed normal T cell counts but low B and NK cell counts.

**Table 2:** Published reports of XLP1 cases with relative longevity (age >40 years).

Age at clinical onset (years)	Age at death or report (years)	Status at publication	Clinical manifestations (age in years)	IVIG	*SH2D1A* mutation (gene)	SAP mutation (protein)	SAP expression	Genetic reversion^#^	Reference
49^*∗*^	51	Deceased	Clinical EBV (18) CNS vasculitis (49)	−	c.35 G > T	p.Ser12Ile	Premature degradation but functional	Unknown	Blackburn et al. [20]

59^*∗*^	65	Deceased	Diffuse large B-cell lymphoma (64)	−	c.35 G > T	p.Ser12Ile	Premature degradation but functional	Unknown	Blackburn et al. [20]

41	44	Alive	Cerebral B-cell lymphoma (41) Hypogammaglobulinemia (43)	+	c.462 C > T	p.Arg55X	Not done	Unknown	Hervier et al. [21]

17	45	Alive	Clinical EBV (17) Hypogammaglobulinemia (26)	+	c.259 T > C	p.Phe87Ser	Deficient	Yes	Palendira et al. [22]

10	49	Deceased	B-cell lymphoma (10) Clinical EBV (19) Hypogammaglobulinemia (19) Meningitis (19) Lymphoproliferation (40) HLH (49)	−	c.161 A > G	p.Tyr54Cys	Deficient	Yes	Palendira et al. [22]

20	52	Alive	Clinical EBV (20) Hypogammaglobulinemia (21)	+	c.259 T > C	p.Phe87Ser	Deficient	Yes	Palendira et al. [22]

6	44	Deceased	Lymphoid interstitial pneumonia (6) Clinical EBV (12) Hypogammaglobulinemia (12) CNS vasculitis (13, 43) Hodgkin lymphoma (44)	+	Deletion	No protein	No expression	No	This study

^*∗*^The two cases from Blackburn et al. [20] were brothers. ^#^Genetic reversion is defined as a secondary mutation that counteracts the effects of the primary mutation, thereby reversing the phenotype back to wild type.

## Data Availability

All immunology assay data are available upon request.

## References

[1] Purtilo D. T., Grierson H. L. (1991). Methods of detection of new families with X-linked lymphoproliferative disease. *Cancer Genetics and Cytogenetics*.

[2] Shlapatska L. M., Mikhalap S. V., Berdova A. G. (2001). CD150 association with either the SH2-containing inositol phosphatase or the SH2-containing protein tyrosine phosphatase is regulated by the adaptor protein SH2D1A. *The Journal of Immunology*.

[3] Panchal N., Booth C., Cannons J. L., Schwartzberg P. L. (2018). X-linked lymphoproliferative disease type 1: a clinical and molecular perspective. *Frontiers in Immunology*.

[4] Meazza R., Tuberosa C., Cetica V. (2014). Diagnosing XLP1 in patients with hemophagocytic lymphohistiocytosis. *Journal of Allergy and Clinical Immunology*.

[5] Tabata Y., Villanueva J., Lee S. M. (2005). Rapid detection of intracellular SH2D1A protein in cytotoxic lymphocytes from patients with X-linked lymphoproliferative disease and their family members. *Blood*.

[6] Pasquier B., Yin L., Fondanèche M.-C. (2005). Defective NKT cell development in mice and humans lacking the adapter SAP, the X-linked lymphoproliferative syndrome gene product. *Journal of Experimental Medicine*.

[7] Nichols K. E., Hom J., Gong S.-Y. (2005). Regulation of NKT cell development by SAP, the protein defective in XLP. *Nature Medicine*.

[8] Purtilo D., Yang J. S., Cassel C. (1975). X-linked recessive progressive combined variable immunodeficiency (duncan’s disease). *The Lancet*.

[9] Williams L. L., Brenner M. K., Krance R. A. (Sep. 1993). Correction of Duncan’s syndrome by allogeneic bone marrow transplantation. *The Lancet*.

[10] Booth C., Gilmour K. C., Veys P. (2011). X-linked lymphoproliferative disease due to SAP/SH2D1A deficiency: A multicenter study on the manifestations, management and outcome of the disease. *Blood*.

[11] Rogers B. B., Browning I., Rosenblatt H. (1992). A familial lymphoproliferative disorder presenting with primary pulmonary manifestations. *American Review of Respiratory Disease*.

[12] Nademi Z., Radwan N., Rao K., Gilmour K., Worth A., Booth C. (2019). Different phenotypic presentations of X-linked lymphoproliferative disease in siblings with identical mutations. *Journal of Clinical Immunology*.

[13] Kanegane H., Yang X., Zhao M. (2012). Clinical features and outcome of X-linked lymphoproliferative syndrome type 1 (SAP deficiency) in Japan identified by the combination of flow cytometric assay and genetic analysis. *Pediatric Allergy and Immunology*.

[14] King T. E. (1999). Pulmonary lymphomatoid granulomatosis. *Advances in Anatomic Pathology*.

[15] Young L. R. (2020). Lymphocytic interstitial pneumonia in children. https://www.uptodate.com/contents/lymphocytic-interstitial-pneumonia-in-children?search=xlp1&topicRef=4361&source=see_link#H3.

[16] Kanegane H., Ito Y., Ohshima K. (2005). X-linked lymphoproliferative syndrome presenting with systemic lymphocytic vasculitis. *American Journal of Hematology*.

[17] Rougemont A.-L., Fournet J.-C., Martin S. R. (2008). Chronic active gastritis in X-linked lymphoproliferative disease. *The American Journal of Surgical Pathology*.

[18] Talaat K. R., Rothman J. A., Cohen J. I. (2009). Lymphocytic vasculitis involving the central nervous system occurs in patients with X-linked lymphoproliferative disease in the absence of Epstein-Barr virus infection. *Pediatric Blood & Cancer*.

[19] Mejstříková E. (2012). Skin lesions in a boy with X-linked lymphoproliferative disorder: Comparison of 5 SH2D1A deletion cases. *Pediatrics*.

[20] Blackburn P. R., Lin W.-L., Miller D. A. (2019). X-linked lymphoproliferative syndrome presenting as adult-onset multi-infarct dementia. *Journal of Neuropathology & Experimental Neurology*.

[21] Hervier B., Latour S., Loussouarn D. (2010). An atypical case of X-linked lymphoproliferative disease revealed as a late cerebral lymphoma. *Journal of Neuroimmunology*.

[22] Palendira U., Low C., Bell A. I. (2012). Expansion of somatically reverted memory CD8+ T cells in patients with X-linked lymphoproliferative disease caused by selective pressure from Epstein-Barr virus. *The Journal of Experimental Medicine*.

[23] Jin Y.-Y., Zhou W., Tian Z.-Q., Chen T.-X. (2016). Variable clinical phenotypes of X-linked lymphoproliferative syndrome in China: Report of five cases with three novel mutations and review of the literature. *Human Immunology*.

[24] Zhu J., Zhang Y., Zhen Z.-J. (2013). Lymphoma and cerebral vasculitis in association with X-linked lymphoproliferative disease. *Chinese Journal of Cancer*.

[25] Xu T., Zhao Q., Li W. (2020). X-linked lymphoproliferative syndrome in mainland China: review of clinical, genetic, and immunological characteristic. *European Journal of Pediatrics*.

[26] Chadha S. N., Amrol D. (2010). X-linked lymphoproliferative disease presenting as pancytopenia in a 10-month-old boy. *Case Reports in Medicine*.

[27] Sun J., Ying W., Liu D. (2013). Clinical and genetic features of 5 chinese patients with X-linked lymphoproliferative syndrome. *Scandinavian Journal of Immunology*.

